# Comparison of Continuous and Pulsating Water Jet during Piercing of Ductile Material

**DOI:** 10.3390/ma16093558

**Published:** 2023-05-06

**Authors:** Akash Nag, Madhulika Srivastava, Jana Petrů, Petra Váňová, Ashish Kumar Srivastava, Sergej Hloch

**Affiliations:** 1 Faculty of Mechanical Engineering, VŠB—Technical University of Ostrava, 708 00 Ostrava, Czech Republic; 2 Department of Mechanical Engineering, Amrita School of Engineering, Amrita Vishwa Vidyapeetham, Chennai 601103, India; s_madhulika@ch.amrita.edu; 3 Faculty of Material Science and Technology, VŠB—Technical University of Ostrava, 708 00 Ostrava, Czech Republic; petra.vanova@vsb.cz; 4 Department of Mechanical Engineering, G. L. Bajaj Institute of Technology and Management, Greater Noida 201306, India; 5 Faculty of Manufacturing Technologies, Technical University of Kosice with a Seat in Prešov, 080 01 Prešov, Slovakia

**Keywords:** erosion, continuous and pulsating water jet, erosion depth, microhardness

## Abstract

More efficient ways to process materials are constantly being sought, even in the case of continuous water flow technology, which acts on materials mainly by stagnant pressure. An alternative method is an ultrasound-stimulated pulsating water jet, the basis of which is the repeated use of impact pressure, which reduces the time interval for mechanical relaxation. This article focuses on a comparative study from the point of view of water mass flow rate on material penetration and its integrity. Relatively low pressures (*p* = 20, 30, and 40 MPa) with varying nozzle diameters (*d* = 0.4 and 0.6 mm) were used to identify the effectiveness of the pulsating water jet. The time exposure of the jet at a fixed place was varied from t = 0.5 to 5 s for each experimental condition. The results showed that with an increase in the pressure and diameter values, the disintegration depth increased. In addition, the surface topography and morphology images showed signs of ductile erosion in the form of erosion pits, upheaved surfaces, and crater formation. The microhardness study showed an increase of 10% subsurface microhardness after the action of the pulsating water jet as compared to the original material.

## 1. Introduction

Water jets have been extensively used for the disintegration and surface treatment of ductile materials over the last few decades due to low reaction forces and high efficiency [1,2,3]. To increase the erosive effect of water jets, several modifications have been developed, including the cavitation water jet [4], abrasive water jet (AWJ) [5,6], and pulsating water jet (PWJ) [7,8]. A PWJ, which is a non-continuous stream, is composed of a series of discrete water clusters acting one after another at short time intervals [9]. It causes fatigue of the material due to the so-called “Hammer Effect” [10,11]. This effect has potential use in material disintegration due to high energy intensity, simple machinery, and minimal wear characteristics of technological equipment components [12,13]. Despite the significant advances achieved in the last decade in the research of water jet technology, researchers pay considerable attention to improving the performance of the technology, adapting to environmental requirements, and rationalizing the economic point of view. The aim is to disintegrate materials without the need for abrasive material [14]. The absence of abrasive mass flow (*m_a_*) reduces the total kinetic energy *E_k_*. Research teams have attempted to compensate for this loss by generating water jets at speeds above values of *v* ≥ 1000 m/s. Such water jets need to be generated at ultra-high pressures [15]. There are well-known attempts to disintegrate metallic materials with pure water without an abrasive at pressures of *p* = 690 MPa [9] and *p* = 900 MPa [16]. The disadvantage is that at such high pressures (690–900 MPa), commercially available components are not able to withstand the load, which has an adverse effect on the service life and reliable operation of the technological process. A potential solution to the problem is the repeated use of the impact pressure *p_i_* during the collision of the liquid with the surface of the material [17,18]. This phenomenon is known from works published at the beginning of the 20th century [11], which have clarified the so-called water hammer effect of the non-periodic action of drops on rotating parts of turbines, causing erosion. The pressure wave speed for incompressible liquids was found by the authors of [11,19]. In [20], the authors stated that Johannes von Kries later published the theory for the water hammer and derived the “Joukowsky” relation. The interaction of liquid in the form of droplets or the water column was analyzed by Joukowsky. He pointed out the importance of compressional waves in a liquid droplet of mass *m* due to an external force *F* acting on the solid surface of the liquid droplet or stream per unit area *A*—considered the collision epicenter. In his work, he considered the compressibility of the liquid. He proved that the impact pressure *p_i_* is higher than the stable (stagnation) flow pressure *p_s_* at the same speed *v*. It was noted that high dynamic pressures can occur in a liquid due to normal liquid compression shock, which introduces a sound pressure limit. In addition, the manifestations of metal erosion were discussed, and this erosion is still considered a serious problem that causes material damage due to liquid impact. When studying the influence of the hardness of metals (pure aluminum, aluminum alloy, brass, mild steel) on erosion rate, the erosion rate was observed to decreases with the increase in hardness and liquid film thickness. In addition, an empirical relation between the material hardness, liquid film thickness, droplet velocity, and local volume flux was developed to predict the erosion. The exposure of the Ti-6Al-4V (rolled) samples with the high-speed water droplet erosion (WDE) [21] showed the presence of surface and sub-surface damage. The microstructural morphology of the eroded craters showed the progressive mechanism of erosion along with the nucleation of cracks, crack propagation, and removal of large fragments of material. The mechanism of the Ti-6Al-4V was also explored under the influence of impact speed (150–350 m/s), which revealed that at a higher impact speed, the erosion rates reached a maximum and erosion initiation time was faster. The microstructural analysis (SEM) showed the presence of microcracks, pits, and upheaved features at the advanced stages of erosion. The most significant means of erosion during the advanced stage of WDE was hydraulic penetration. Moreover, the effect of the initial surface roughness of martensitic stainless steel and Ti-6Al-4V on the erosion behavior was also investigated. It was observed that the length of the incubation zone was influenced by the initial surface quality of the material due to the presence of the surface asperities/irregularities. The investigation of an ex-service stainless steel turbine blade [20] showed the presence of erosion features (pits, cracks, asperities, depressions) which were comparable to the laboratory test rig results. It was also observed that the twist angle of the blade was a significant parameter that influenced the WDE phenomenon. Although many studies have focused on solving the problems related to WDE, a lack of understanding remains regarding the possibility of treating this serious issue. 

For fortified interruption of the continuous water flow, Foldyna and Svehla [18] developed an ultrasonic PWJ method with a variable acoustic chamber length *lc* (mm). The variation in *lc* (mm) allows tuning of the resonant frequency and the output power. In the acoustic chamber, the ultrasonic sonotrode creates pressure fluctuations in pressurized water of 10–100 MPa supplied by the pump. The sonotrode generates standing waves inside the acoustic chamber, which are pushed toward the exit of the nozzle, through the narrowing space inside the acoustic chamber. The amplitude of the standing waves increases toward the outlet of the nozzle due to its converging shape. After exiting the nozzle, these pressure fluctuations turn into velocity fluctuations, which manifest themselves at a certain distance from the nozzle exit [22]. Changes in axial velocity cause a forced disintegration of the nozzle to form water clusters of liquid droplets [23]. A series of water pulses periodically hits the surface and erodes the material in different erosion modes [24]. These modes have been explained parametrically [25,26]. Some works [27,28,29] focusing on the investigation of the technology of ultrasonically excited water flow have, primarily investigated the technological parameters or were also oriented toward the characterization of the material. These works were based on the distribution of water droplets in a moving nozzle, using the traverse speed with different velocity. 

In the current study, the authors focus on a statically acting concentrated pulsating water jet with a time-varying exposure. The main goal was to determine the effect of the nozzle diameter along with the effect of the supply pressure on the erosion phenomenon, which is not fully addressed even in the classical water hammer theory. This is the preliminary study conducted where the supply pressure was changed from *p* = 20 MPa to 40 MPa and the nozzle diameter from *d* = 0.4 to 0.6 mm. The erosion phenomenon was analyzed on the basis of the obtained surface topography, morphology, and microhardness results.

## 2. Materials and Methods

The aluminum alloy AW-6060 was used in the current experiments to observe the effect of time exposure along with nozzle diameter and supply pressure variations. Aluminum alloys are used in various industries and applications, mainly in automobile industries, aviation, and aerospace for their light weight and superior mechanical properties. During their service life, aluminum components are sometimes exposed to water droplets in the form of rain or other forms. This interaction of the water droplets reduces the working efficiency and lifetime of the components. Therefore, it is important to understand the interaction phenomenon of the water droplets with the aluminum workpiece. In addition, due to the ductility of the aluminum alloy, the disintegration grooves generated by the action of PWJ are clearly visible and can be linked to the input parameters levels. An aluminum plate with dimensions of 250 mm in length, 100 mm in width, and 5 mm in thickness was used in this experiment. An ultrasonic pulsating water jet device developed and available at the Institute of Geonics at the CAS, Czech Republic was used for the experiment. The setup consists of a high-pressure pump capable of operating at a maximum pressure of *p* = 160 MPa with a flow rate of *Q* = 67 L/min. This high-pressure water entered the high-pressure acoustic chamber present inside the pulsating head. Ultrasonic oscillations were generated using an ultrasonic generator with a spatial frequency of *f* = 20 kHz. The ultrasonic signals were transmitted to the piezoelectric ceramics via cables which were attached to the mechanical sonotrode, which acted as a transducer to transform the ultrasonic energy into mechanical energy. When they come into contact with a high-pressure water stream, these ultrasonic vibrations generated pressure fluctuations inside the acoustic chamber. These pressure fluctuations travelled in the form of standing waves through the internal waveguide to reach the nozzle exit. When exiting the nozzle, the pressure fluctuations transformed into velocity fluctuations resulting in different a velocity amplitude for different sections of the jet. This jet eventually broke into a series of fast- and slow-moving sections of water clusters, resulting in the formation of a pulsating water jet. The trajectory and positioning of the jet are controlled by fitting the pulsating head on an ABB robotic arm which could control and maneuver the entire pulsating head over the material at the desired position. 

**Figure 1:**
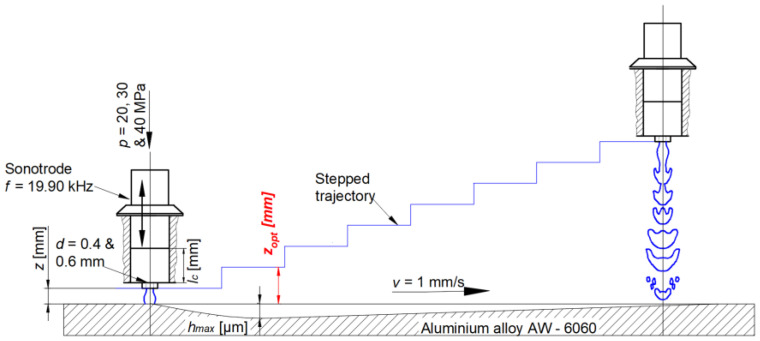
Schematic representation of the methodology for obtaining the optimal standoff distance.

**Figure 2:**
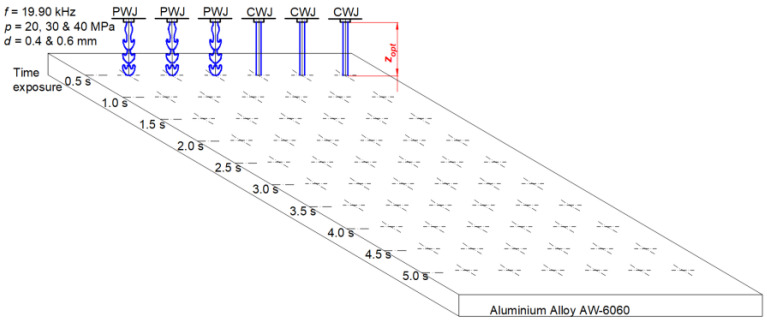
Experimental scheme to investigate the interaction of PWJ and CWJ at different pressure levels (20, 30, and 40 MPa) and nozzle diameters (0.4 and 0.6 mm) with time exposure (0.5–5.0 s).

In this experiment, the influence of supply pressure, nozzle diameter, and time exposure on the disintegration depth of the aluminum sample was investigated. Three levels of supply pressure, i.e., *p* = 20, 30, and 40 MPa, along with two levels of nozzle diameter, *d* = 0.4 and 0.6 mm, were selected based on pilot experiments which focused on the stability of the jet with uniform flow. These parameters were also selected to explore the potential of PWJ even at such low pressures and volumetric flow rates. Another reason to select these parameters was to observe exactly when the erosion in the material starts, i.e., to identify the pre-incubation and incubation stage, which is not possible with a larger volumetric flow rates with the selected material. The time exposure for each combination of parameters was varied from 0.5 s to 5 s in intervals of 0.5 s. The experimental conditions are listed in Table 1. However, prior to conducting the main experiments, tuning of the whole ultrasonic system was required and was performed by changing the length of the acoustic chamber from *lc* = 0 to 25 mm. The goal was to achieve the resonant frequency in the impedance regime. In practice, it corresponds to the chamber length setting having the lowest frequency with the appropriate ultrasonic output power. The steps of tuning the technology have been presented in detail previously [30]. The optimal chamber lengths were selected for each combination of the nozzle diameter and supply pressure and are mentioned in Table 1. After optimizing the acoustic chamber length, the optimal standoff distance was obtained. For obtaining the optimal standoff distance, the distance between the nozzle exit and the material surface was varied from 1 mm to 60 mm (Figure 1). A step trajectory was followed with horizontal movement of 10 mm and a vertical step height of 1 mm. This procedure was also repeated for each combination of nozzle diameter and supply pressure. The optimal values of standoff distances for each operating condition are mentioned in Table 1. Therefore, after achieving the optimal values for the acoustic chamber length and standoff distance, the main experiments were carried out following the experimental schematic diagram shown in Figure 2. In addition, for the comparison of the efficiency of the PWJ over the continuous water jet (CWJ), all experimental runs were repeated with CWJ without any pulsations. The impact pressure and stagnation pressure induced by the PWJ and CWJ into the material during its interaction with the aluminum alloy was also calculated using the impact pressure equation (*p_i_*) in Equation (1), given by de Haller, and the stagnation pressure equation (*p_s_*) in Equation (2), where *ρ_l_* and *ρ_s_* are the densities of the interacting liquid and solid, respectively, *c_l_* and *c_s_* are shock wave velocities for the liquid and solid, and *v* is the velocity of the impacting jet.

(1) $$p_{i}=\frac{v\rho_{l}c_{l}\rho_{s}c_{s}}{\rho_{l}c_{l}+\rho_{s}c_{s}}$$

(2) $$p_{s}=\frac{1}{2}\rho_{l}v^{2}$$

Three repetitions of each experimental run were performed to obtain better statistically valid results. After the experimental runs, the disintegration marks were scanned using an Alicona Infinite Focus G5 optical microscope to measure the extent of disintegration damage corresponding to different input parameters. This method of measurement of erosion depth was selected due to its accuracy, speed, and versatility. The scanned images were post-processed using built-in analysis software to calculate the disintegration depth. 

Surface integrity studies were carried out in selected samples generated under specific conditions to understand the erosion phenomenon in more detail. The grooves formed by exposure of the jet with time exposure *t* = 0.5 s with nozzle diameter *d* = 0.4 and 0.6 mm along with supply pressure *p* = 20 and 40 MPa were selected. In addition, for comparison of the efficiency of the PWJ over CWJ, the sample exposed to a time exposure *t* = 5 s using the nozzle diameter *d* = 0.6 mm and supply pressure *p* = 40 MPa was also selected for surface integrity studies. The surface topography and cross-sectional microstructure of the impacted region was observed using an Olympus IX70 light microscope in the state after polishing. The surface morphology of the generated erosion crater was examined using a JEOL 6490LV scanning electron microscope (SEM) in secondary electron mode (SE). The subsurface microhardness of the selected samples was measured along the cross-section of the generated crater. The hardness was measured on a DuraScan G5 hardness tester according to the Vickers method under a load of 10 g. The distance between two indents was kept at 60 µm from the tip of the crater until 1 mm and 200 µm for depth from 1 to 1.6 mm below the crater. The microhardness testing was carried out according to ČSN EN ISO 6507-1 (Metallic materials—Vickers hardness test—Part 1: Test method).

## 3. Result and Discussion

### 3.1. Influence of Time Exposure on Disintegration Depth

**Figure 3:**
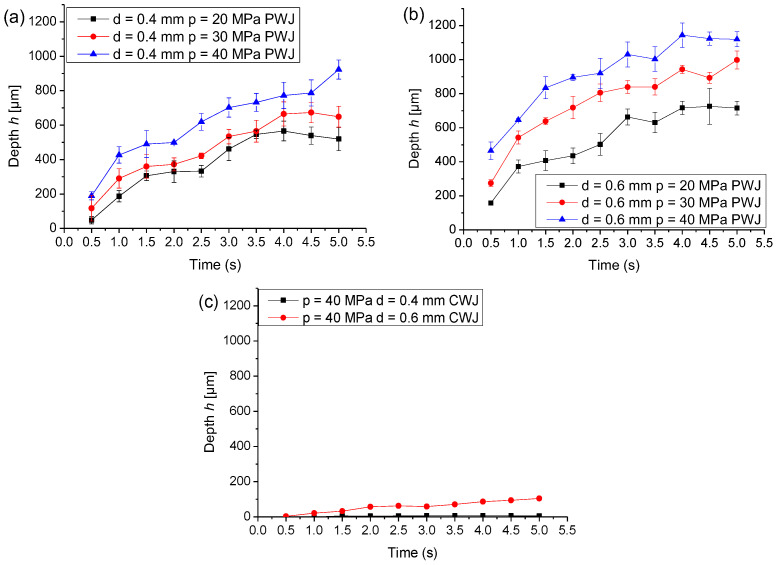
Influence of supply pressure (*p* = 20, 30, and 40 MPa), time exposure (*t* = 0.5–5.0 s), and nozzle diameter (**a**) *d* = 0.4, (**b**) *d* = 0.6 mm for PWJ and (**c**) *p* = 40 MPa with *d* = 0.4 and 0.6 mm for CWJ on disintegration depth.

Figure 3 depicts the influence of input parameters on disintegration depth. For the nozzle diameter *d* = 0.4 mm, the overall disintegration depth increased for all time exposures with the increase in the supply pressure. The mechanism of material erosion started with surface roughening of the aluminum material surface due to repetitive impingements of the PWJ inducing impact pressure followed by shearing of the surface by the lateral jetting. The combined effect straied the material beyond the ultimate strength of the material, and the first site of microcracks appeared. This crack further propagated and led to separation of a volume of material, forming micropits. These shallow surface pits or depressions further deepened with subsequent droplet impingements. In addition, the material heterogeneity played a role in the initial yielding of the material after the interaction with the PWJ. Lateral jetting, which occurred at the onset of the water hammer effect, also generates surface asperities, which in turn acted as potential sites for stress concentration and the initiation of fatigue cracks and their propagation. These factors led considerable damage of the material in the form of ductile failure, and detectable erosion was observed. With the increase in supply pressure, along with the increase in the volumetric flow rate and hydraulic power, the magnitude of impact pressure (Table 1) also increased, which caused larger material deformation with less time exposure or a lower number of impingements. Therefore, a deeper disintegration groove was observed in general for larger supply pressure as compared to lower pressure, keeping all other parameters constant. For instance, at a constant time exposure of *t* = 0.5 s, the disintegration depth increased from *h* = 47.91 µm to 188.90 µm for an increase in the supply pressure from *p* = 20 to 40 MPa, respectively.

For a constant hydraulic parameter, such as *d* = 0.4 mm and *p* = 20 MPa, with an increase in time exposure from *t* = 0.5 to 5 s, the disintegration depth increased from *h* = 47.91 µm to 548.85 µm. This increase in disintegration depth by 11-fold was due to an increase in the number of impingements from 9950 impacts to 99,500 impacts from *t* = 0.5 to 5 s, respectively. An accelerated increase in the disintegration depth corresponding to time exposure was observed until 4 s. After 4 s, the disintegration depth corresponding to the further increase in the time exposure became constant due to the presence of water already in the crater generated, which resisted the effective interaction of the subsequent water interacting in the same place. Thus, the rate of disintegration depth became either constant or slightly declined at higher time exposure. For the increase in the pressure from 20 to 30 MPa, an overall increase in the disintegration values was measured. At the supply pressure of *p* = 30 MPa, the disintegration depth increased from 117.05 µm to 648.75 µm for an increase in time exposure from *t* = 0.5 to 5.0 s, respectively. In addition, for this specific flow, the rate of increase in the erosion depth showed an accelerated trend until 4 s and then the depth remained constant until 5 s. For the supply pressure *p* = 40 MPa, the disintegration depth varied from *h* = 188.90 µm to 922.85 µm for the time exposure from *t* = 0.5 to 5.0 s, respectively. However, for these specific flow condition, the disintegration depth increased for the entire exposure time (0.5–5.0 s), which was different from the previous experiments. For the time exposure of *t* = 5 s, the disintegration depth increased 1.68 times for *p* = 40 MPa as compared to 20 MPa due to the increase in the hydraulic power of the jet from *P* = 272 W to 768 W. Overall, with the increase in the supply pressure and time exposure, the disintegration depth increased, and the magnitude of the disintegration depended on the level of the parameters. For the nozzle diameter *d* = 0.6 mm, the disintegration depth deepened for all pressure values and time exposures as compared to the disintegration depth measured with *d* = 0.4 mm. For *p* = 20 MPa and *d* = 0.6 mm, *h* = 158.54 µm as compared to *h* = 47.91 µm for *p* = 20 MPa and *d* = 0.4 mm at *t* = 0.5 s. This increase in the depth values can be attributed to the increase in the hydraulic power with *d* = 0.6 mm (*P* = 611, 1123, and 1729 W for *p* = 20, 30, and 40 MPa, respectively) as compared to *d* = 0.4 mm (*P* = 272, 499, and 768 W for *p* = 20, 30, and 40 MPa, respectively). However, the magnitude of the impact pressure calculated remained the same even with the increased nozzle diameter. Therefore, the current study shows the importance of both the droplet size and supply pressure on the material erosion. With an increase in the time exposure from *t* = 0.5 s to 5 s, the disintegration depth increased from *h* = 158.54 µm to 715.90 µm. The erosion started immediately from the exposure time *t* = 0.5 s, but the rate of erosion became nearly constant after 4 s of time exposure as observed with the 0.4 mm nozzle diameter. With the increase in the supply pressure *p* = 30 MPa, the disintegration depth also increased. This is attributed to an increase in the hydraulic power from 611 W to 1123 W for *p* = 20 to 30 MPa, respectively. The disintegration depth changed from 274.48 µm to 997.95 µm for the time exposure from *t* = 0.5 to 5 s. With the further increase in the supply pressure to *p* = 40 MPa, the disintegration depth varied from *h* = 464.91 µm to 1119.85 µm for the time exposure from *t* = 0.5 s to 5.0 s. The maximum disintegration depth of *h* = 1143.93 µm was obtained at time exposure *t* = 4.0 s with the nozzle diameter *d* = 0.6 mm and supply pressure *p* = 40 MPa. This magnitude of disintegration was obtained due to the higher hydraulic power *P* = 1729 W along with the longer exposure time *t* = 4.0 s. The above discussed section shows the reach of disintegration depth and its influence on input parameters of the PWJ technology.

To compare the efficiency of PWJ with CWJ, experiments with CWJ with each combination of parameters were carried out. However, measurable erosion traces were detectable with only two specific combinations of input parameters, i.e., *p* = 40 MPa with *d* = 0.4 mm and 0.6 mm. The disintegration depth measured during the impact of CWJ with the material at different time exposure is shown in Figure 3. For *d* = 0.4 mm, the disintegration depth increased from 0 to 5.00 µm for time exposure from *t* = 0.5 s to 5.0 s. The depth obtained can also be considered as surface roughness of the impacted surface. For same technological input, PWJ generated a depth of 922.85 µm as compared to 5.00 µm generated by CWJ for time exposure *t* = 5.0 s with *d* = 0.4 mm and *p* = 40 MPa. This large enhancement in the efficiency of the PWJ was due to the repetitive impingements of water droplets over a fixed area. These impacts induced impact pressure in form of compressive stresses, which propagates through the material and is followed by radial outflow, which induced shear stresses into the material. For PWJ, the impact pressure for *p* = 20, 30, and 40 MPa was 242.79, 297.35, and 343.35 MPa, respectively, as compared to stagnation pressure of 16.23, 23.35, and 32.46 MPa for CWJ. This large difference in the magnitude of the induced stresses resulted in higher material disintegration for PWJ, even with lower input technological parameters. For *d* = 0.6 mm and *p* = 40 MPa, the disintegration depth varied from 3.13 µm to 104.27 µm for a time exposure from *t* = 0.5 s to 5.0 s, respectively. For comparison, PWJ with *d* = 0.6 mm, *p* = 40 MPa, and *t* = 5.0 s generated disintegration depth of 1119.85 µm as compared to 104.27 µm for CWJ, keeping all input parameters the same. The reason for this change in the erosion behavior is explained above. Therefore, the efficiency of PWJ for disintegration application is much higher than conventional water jet variants.

### 3.2. Microstructural Morphology and Topography

**Figure 4:**
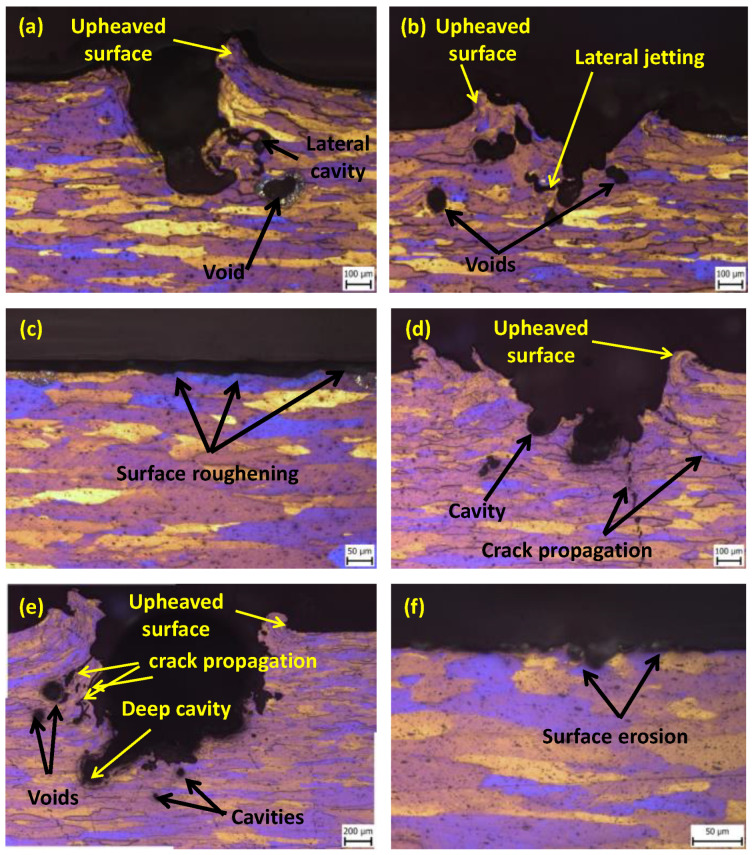
Cross-sectional topography of the region disintegrated by PWJ using (**a**) *d* = 0.4 mm, *t* = 0.5 s, *p* = 20 MPa; (**b**) *d* = 0.4 mm, *t* = 0.5 s, *p* = 40 MPa; (**c**) *d* = 0.6 mm, *t* = 0.5 s, *p* = 20 MPa; (**d**) *d* = 0.6 mm, *t* = 0.5 s, *p* = 40 MPa; (**e**) *d* = 0.6 mm, *t* = 5 s, *p* = 40 MPa; and (**f**) *d* = 0.6 mm, *t* = 5 s, *p* = 40 MPa (CWJ).

The cross-sectional section of the samples exposed to the impacts at *p* = 20 MPa and 40 MPa for the duration 0.5 s, using a nozzle of a diameter *d* = 0.4 mm and 0.6 mm, was observed under an optical microscope. The sample treated at *p* = 20 MPa (Figure 4a) showed the formation of an erosion pit of a depth of 84 µm. This feature is attributed to the hydraulic penetration of the repeated water clusters into the surface asperities of the material and the radial outflow of the jet. The formation of the tapered tunnel shown in Figure 4b was the result of the interruption of the incoming jet with the surface, which prevented further penetration of the jet. The induced shear stress exceeded the yield strength of the material and was responsible for the formation of an upheaved surface. Similar features were also reported during the water jet erosion of the rolled Ti-6Al-4V [17]. The formation of a deeper lateral sub-tunnel (310 µm) was observed in the samples treated at a higher pressure of 40 MPa in comparison to lower pressure of 20 MPa (84 µm) due to the difference in the hydraulic power of the jet from 768.50 W to 271.71 W.

The samples treated with the nozzle diameter of *d* = 0.6 mm at 20 MPa (Figure 4c) showed surface roughening on the impacted surface. This feature is attributed to the occurrence of the lateral outflow of the jet (radially) after the initial impact. However, when increasing the pressure to 40 MPa (Figure 4d), an eroded groove with a depth of 372 µm, along with the presence of a cavity and upheaved surface were observed due to the shear stress caused by the peripheral lateral outflow of the jet. In addition to these features, the transgranular and intergranular cracks along the surface of the tunnel were also visible, which can be attributed to the concentration of stress in a particular confined area, that exceeded the ultimate limit of the material. These cracks further propagated through the material surface and connected with other surface cracks, leading to the removal of a larger volume of material. The erosion depth for the nozzle diameter 0.6 mm is greater than for the nozzle diameter 0.4 mm due to the increase in the flow rate from 0.82 L/min to 1.83 L/min. On comparing the effect of CWJ and PWJ, it was observed that the sample treated by PWJ showed the presence of an erosion tunnel along with voids, cavities [31], and cracks (Figure 4e) while the sample treated with CWJ (Figure 4f) shows small erosion pits in the near surface regions. The difference in the features is due to the predominance of the stagnation pressure over the impact pressure induced by CWJ.

**Figure 5:**
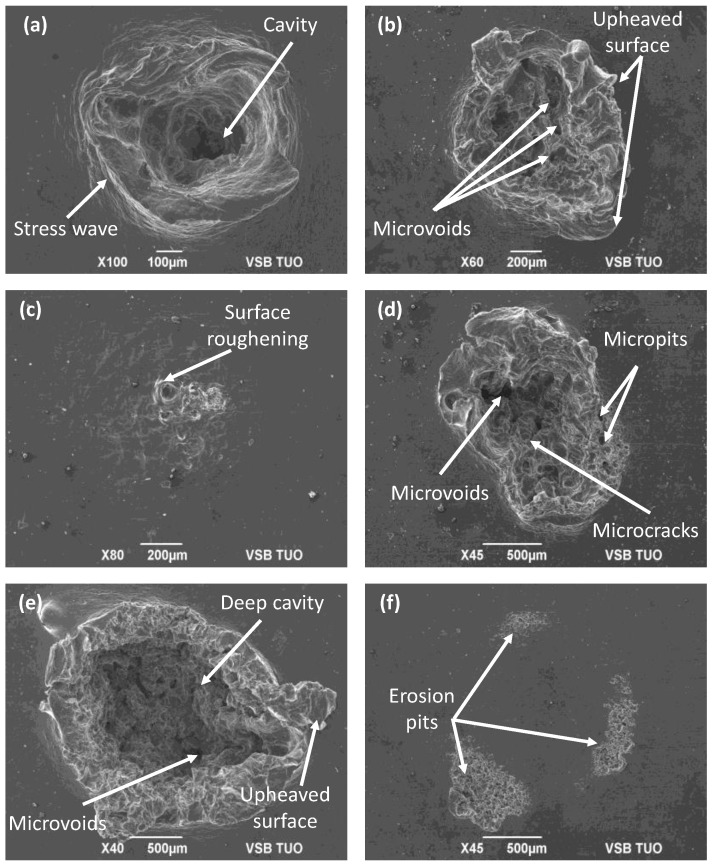
Surface morphology of the region generated by PWJ using (**a**) *d* = 0.4 mm, *t* = 0.5 s, *p* = 20 MPa; (**b**) *d* = 0.4 mm, *t* = 0.5 s, *p* = 40 MPa; (**c**) *d* = 0.6 mm, *t* = 0.5 s, *p* = 20 MPa; (**d**) *d* = 0.6 mm, *t* = 0.5 s, *p* = 40 MPa; (**e**) *d* = 0.6 mm, *t* = 5 s, *p* = 40 MPa; and (**f**) *d* = 0.6 mm, *t* = 5 s, *p* = 40 MPa (CWJ).

The surface morphology of the impacted region is shown in Figure 5. At the pressure of 20 MPa (Figure 5a), the presence of a cavity was observed in the impacted region. This effect was the result of the hammering caused by the distinct clusters of the water jet. 

The microstructural distortion due to the lateral and longitudinal stress propagation in the impacted zone led to the formation of stress waves on the periphery of the impacted zone. When increasing the pressure to 40 MPa (Figure 5b), the presence of microvoids was observed in the impacted region due to the microtunnelling. In addtion, the lateral outflow of the jet was responsible for the formation of the upheaved surface along the walls of the crater. The difference in erosion features when increasing the pressure was due to the difference in the hydraulic power of the jet. In contrast to this, the samples treated with an increased nozzle diameter *d* = 0.6 mm at *p* = 20 MPa showed surface roughening and, at *p* = 40 MPa, the presence of microcracks, micropits, and microvoids was observed. This variation in the morphology was due to the difference in the flow rates caused by the different nozzle diameters. Furthermore, on comparing the erosion effect of CWJ and PWJ at pressure *p* = 40 MPa and *d* = 0.6 mm, it was observed that the samples treated using CWJ underwent surface roughening with the formation of erosion pits; however, in PWJ, a deeper cavities and microvoids along with the presence of upheaved surfaces, were observed. In the case of PWJ, the predominance of the lateral jetting [32] was responsible for causing these surface features. It also reflects the effectiveness of the PWJ over CWJ in disintegrating ductile materials [25,33].

### 3.3. Microhardness

**Figure 6:**
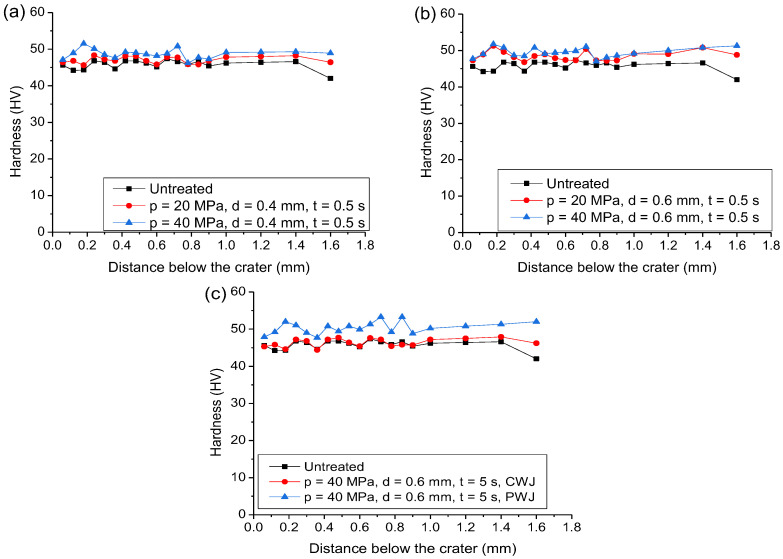
Variation in subsurface microhardness values before and after treatment along the cross-section of the sample, created with (**a**) *p* = 20 and 40 MPa, *d* = 0.4 mm, and *t* = 0.5 s; (**b**) *p* = 20 and 40 MPa, *d* = 0.6 mm, and *t* = 0.5 s; and (**c**) PWJ and CWJ at *p* = 40 MPa, *d* = 0.6 mm, and *t* = 5 s.

The change in the subsurface microhardness of the samples after the action of PWJ was studied in this section. Figure 6a shows the comparison of the microhardness values induced in the sample subsurface impacted with nozzle diameter of *d* = 0.4 mm with a supply pressure of *p* = 20 and 40 MPa. These values were also plotted together with the untreated sample. It is observed that the overall microhardness values did not fluctuate significantly and showed a uniform trend with variation in the distance (0–1.6 mm) from the impacted surfaces for each experimental condition. The microhardness values obtained by the action of PWJ showed higher values (47.07 ± 0.89 HV and 48.74 ± 1.23 HV for *p* = 20 MPa and 40 MPa, respectively) compared to the untreated sample (45.79 ± 1.25 HV). The reason for the increase in the hardness values after the action of PWJ was due to the induction of compressive stresses followed by shear stresses, whichwas due to lateral jetting. These stresses induced in the material led to the accumulation of plastic strain due to plastic deformation, which led to an increase in the microhardness values near the impacted surface. For *p* = 20 MPa, the microhardness values varied in the range of 47.07 ± 0.89 HV for subsurface depth until 1.6 mm below the generated crater. With the increase in the supply pressure to *p* = 40 MPa, the values of microhardness increased to 48.74 ± 1.23 HV. This increase in the hardness values was due to an increase in the velocity of the impacting jet for 40 MPa (*v* = 180.18 m/s) as compared to 20 MPa (*v* = 254.81 m/s). This increase in the impact velocity in turn increased the impact pressure, which was responsible for the induction of higher compressive stresses inside the material. However, the increase in the hardness does not significantly depend on the input parameters due to the very small difference (~25 MPa) between the yield strength and ultimate strength of the aluminum alloy [34]. Therefore, the hardening effect of the subsurface layer is removed with the disintegration of the material surface.

The improvement in the subsurface layer was also observed when exposing the material with a nozzle diameter *d* = 0.6 mm as compared to the original surface (Figure 6b). The original material’s microhardness varied in the range of 45.79 ± 1.25 HV. However, exposing the material to PWJ, the microhardness of the material increased to a range of 48.52 ± 1.28 HV and 49.54 ± 1.27 HV for *p* = 20 and 40 MPa, respectively. As compared to the values resulting after the exposure of PWJ with *d* = 0.4 mm, microhardness values increased more for *d* = 0.6 mm when keeping the same pressure values. The mean microhardness values obtained were 47.07 and 48.74 HV for *p* = 20 and 40 MPa, respectively, for *d* = 0.4 mm, as compared to 48.52 and 49.54 for *p* = 20 and 40 MPa, respectively, for *d* = 0.6 mm. This increase in the microhardness values is attributed to the increase in the hydraulic power of the jet from *P* = 271 and 768 W for *p* = 20 and 40 MPa, respectively, with *d* = 0.4 mm, to *P* = 611 and 1721 W for *p* = 20 and 40 MPa, respectively, with *d* = 0.6 mm. PWJ impacting with higher hydraulic power values induced higher plastic strain inside the material due to larger material deformation and subsurface microstructural changes, which can also be observed in Figure 4. However, it was also observed that the microhardness values for *p* = 20 MPa with *d* = 0.6 mm were found to be lower than *p* = 40 MPa with *d* = 0.4 mm, which confirms the importance of taking into account the hydraulic power of the jet, and shows the importance of nozzle diameter (*P* = 611 W and 768 W for *p* = 20 MPa, *d* = 0.6 mm and *p* = 40 MPa, *d* = 0.4 mm, respectively), rather than only relying on the volumetric flow rate (*Q* = 1.83 L/min and 1.15 L/min for *p* = 20 MPa, *d* = 0.6 mm and *p* = 40 MPa, *d* = 0.4 mm, respectively). In addition, the calculation of impact pressure does not take into the account the importance of the nozzle diameter and only relies on the impacting velocity of the jet, which is a function of the pressure (Table 1). The only parameter which considers the nozzle diameter or the droplet size is in the calculation of the time interval for which the impact pressure is applied on the material surface. However, it can be easily observed that both supply pressure and nozzle size contribute to the overall WDE mechanism. 

For comparing the efficiency of PWJ with CWJ, the sample subsurface microhardness created with extreme parameter levels *d* = 0.6 mm, *p* = 40 MPa, and *t* = 5 s was used. The mean subsurface microhardness values varied from 50.42 ± 1.57 HV for PWJ as compared to 46.38 ± 1.05 HV, which is approximately similar to the material’s original microhardness (45.79 ± 1.25 HV). The increase of approximately 9% in microhardness for PWJ was due to the repetitive induction of impact pressure inside the material as compared to stagnation pressure for CWJ. These repetitive impacts did not allow the material to relax and regain its original structure and changed its structure with minimal energy input. This increase in the microhardness shows the efficiency of the PWJ to be used for peening and surface treatment applications in the required enhancement of the mechanical properties with lower input energy.

## 4. Conclusions

The present study demonstrates a comparison between the continuous and pulsating water jet from the viewpoint of mass flow rate and its integrity during the action of multiple impacts of water drops with time-varying exposure of ductile material. The main findings of the study are as follows:

1. The increase in the supply pressure from *p* = 20 MPa to 40 MPa and time exposure from 0.5 s to 5.0 s caused an increase in the disintegration depth. However, the magnitude of the disintegration depended on the level of the parameters.
2. The surface morphology showed ductile fracture in the form of erosion pits, upheaved surfaces, and cavity formation. Additionally, the cross-sectional microstructural images showed features such as crack propagation, lateral jetting, and surface roughening.
3. The sub-surface microhardness values of the samples treated by PWJ at *p* = 40 MPa showed an increase of 6.44% and an increase of 2.8% at *p* = 20 MPa in comparison with the untreated sample. The effect is attributed to the variation in the velocity of the impacting jet. However, an overall increase of 9% was observed in the case of PWJ in comparison with CWJ.
4. The microhardness values for *p* = 20 MPa with *d* = 0.6 mm were found to be lower than *p* = 40 MPa with *d* = 0.4 mm, due to the predominance of the hydraulic power of the jet, showing the importance of the nozzle diameter selection.
5. Upon comparing the efficiency of the PWJ and CWJ, it was observed that the subsurface microhardness values varied from 50.42 ± 1.57 HV for PWJ as compared to 46.38 ± 1.05 HV, which is equivalent to the material’s initial microhardness (45.79 ± 1.25 HV).

The results presented in this work show the importance of both nozzle diameter and supply pressure in terms of the disintegration magnitude of the selected ductile material, i.e., aluminum AW—6060. It can be concluded that the selection of the input parameter combination depends on the end application; for example, if deeper disintegration is required, than larger nozzle diameter can be used while keeping the pressure value low. However, if precise and limited area disintegration is needed, a lower nozzle diameter and higher pressure levels can be used. This also opens the potential where PWJ can be used as a single technology for peening, disintegration, and surface treatment applications.

## Figures and Tables

**Table 1:** Summary of the experimental conditions.

Sl.No.	Supply Pressure *p* [MPa]	No. of Repetitions *n*	Frequency *f* [kHz]	Acoustic Chamber Length *lc* [mm]	Nozzle Diameter *d* [mm]	Standoff Distance *z* [mm]	Time Exposure *t* [s]	Impact/Stagnation Pressure [MPa]
1	20	3	19.90	16	0.4	20	0.5–5.0	242.79
2	30			16		30		297.35
3	40			18		35		343.35
4	20			18	0.6	25		242.79
5	30			18		46		297.35
6	40			20		52		343.35
7	20	3	CWJ	16	0.4	20	0.5–5.0	16.23
8	30			16		30		24.35
9	40			18		35		32.46
10	20			18	0.6	25		16.23
11	30			18		46		24.35
12	40			20		52		32.46

## Data Availability

The data presented in the paper can be made available on request from the corresponding author.
